# Supplementary material for: Splice-Junction-Based Mapping of Alternative Isoforms in the Human Proteome
Source: Cell Rep. Author manuscript; Available in PMC 2020 Jan 15. (PMC6961840; doi:10.1016/j.celrep.2019.11.026)
Supplement: 2 [file NIHMS1546469-supplement-2.zip › DF1/PXD006675/RightAtrium/RightAtrium_54_EFCAB14_ETPGSNQIIPSPSATSELDNKTHSENLKQMGDR.pdf]

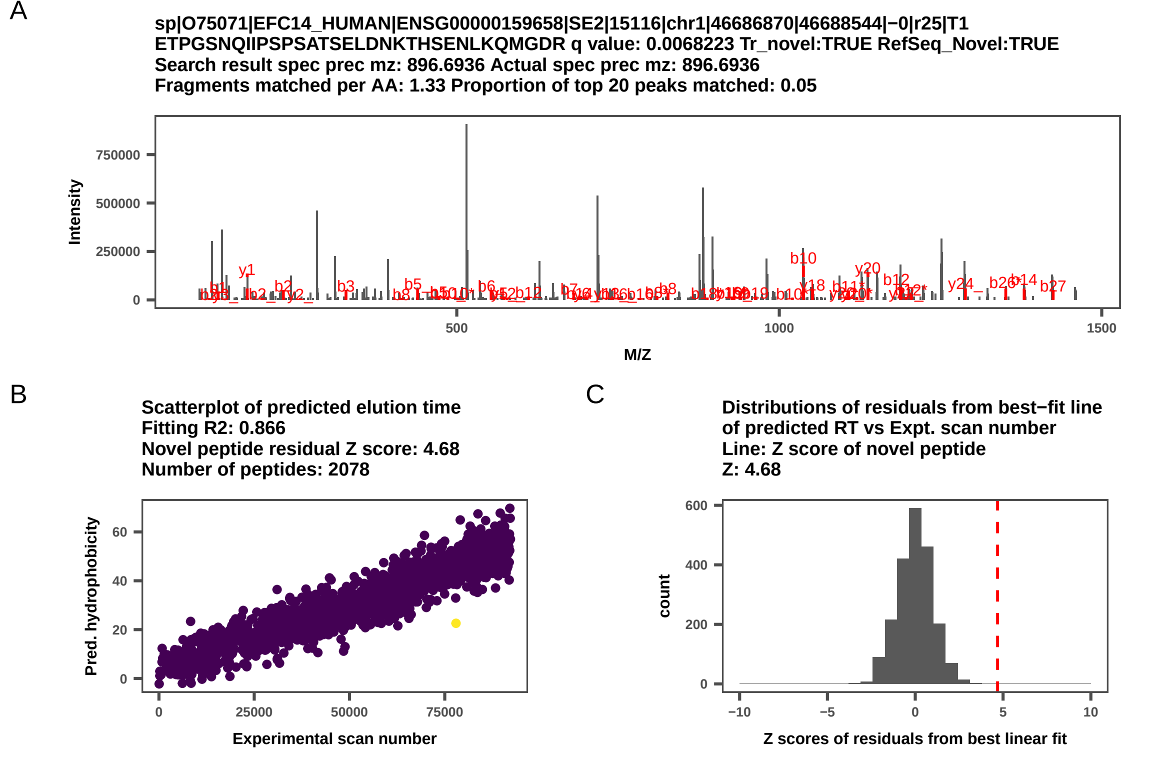

A
sp|O75071|EFC14_HUMAN|ENSG00000159658|SE2|15116|chr1|46686870|46688544|−0|r25|T1
ETPGSNQIIPSPSATSELDNKTHSENLKQMGDR q value: 0.0068223 Tr_novel:TRUE RefSeq_Novel:TRUE
Search result spec prec mz: 896.6936 Actual spec prec mz: 896.6936
Fragments matched per AA: 1.33 Proportion of top 20 peaks matched: 0.05
Intensity
750000
500000
250000
0
500
1000
1500
M/Z
y1
b1
b2
b3
b5
b6
b7
b8
b10
y18
y20
b12
y24
b14
b27
B
Scatterplot of predicted elution time
Fitting R2: 0.866
Novel peptide residual Z score: 4.68
Number of peptides: 2078
Pred. hydrophobicity
60
40
20
0
0
25000
50000
75000
Experimental scan number
C
Distributions of residuals from best−fit line
of predicted RT vs Expt. scan number
Line: Z score of novel peptide
Z: 4.68
count
600
400
200
0
−10
−5
0
5
10
Z scores of residuals from best linear fit
